# Intensive Neurophysiological Rehabilitation System for children with cerebral palsy: a quasi-randomized controlled trial

**DOI:** 10.1186/s12883-023-03216-4

**Published:** 2023-04-20

**Authors:** A Kushnir, O Kachmar

**Affiliations:** Elita Rehabilitation Center, Lviv, Ukraine

**Keywords:** Cerebral palsy, Developmental disabilities, Neurological rehabilitation, Motor skills, Randomized controlled trial [Publication Type]

## Abstract

**Background:**

Recent research indicates that intensive rehabilitation tends to be effective for children with cerebral palsy (CP). Intensive Neurophysiological Rehabilitation System (INRS) is a multi-component approach that combines various interventions and addresses different functional goals.. This study aimed to examine the effectiveness of the INRS treatment in children with bilateral CP.

**Methods:**

In this quasi-randomized controlled study, 48 children with spastic bilateral CP (age 5–12 years, GMFCS Levels I-IV, MACS Levels I-IV) were assigned to an experimental or control group in order they have been enrolled. The experimental group underwent INRS treatment in the tertiary care facility for about four hours daily for ten days and continued routine home treatment for four weeks. After the first evaluation, participants from the control group stayed on the waiting list for four weeks receiving home treatment and then starting the INRS treatment. Thereby, all participants were assessed three times. The primary outcome measure was a Gross Motor Function Measure 66 Item Set (GMFM). The secondary outcome measures included the Jebsen-Taylor Hand Function test, Box and Blocks test, ABILHAND-Kids Questionnaire, Self-care and Mobility domain of the Pediatric Evaluation of Disability Inventory, and the ankle dorsiflexion passive range of motion.

**Results:**

There was a statistically significant increase in the GMFM score after the INRS treatment in both the experimental group (mean difference (MD) 2.0, *P* < 0.01) and control group (MD 1.5, *P* < 0.05), with a large size effect (partial eta squared (η2) = 0.21 and η2 = 0.14). The mean difference between groups during the first study period was 2.89 points (*p* < 0.01) in the GMFM score with a medium effect size (η2 = 0.12). Statistically significant superiority of the INRS treatment over home treatment was also obtained by Jebsen-Taylor Hand Function Test and the Box and Blocks Test in both dominant and non-dominant hands.

**Conclusions:**

The study indicates that the INRS treatment can be beneficial for improving both gross motor functions and hand function in children with bilateral CP. Further longitudinal studies are required to evaluate the effects of the INRS treatment on the participation level of children with CP.

**Trial registration:**

The study protocol was registered on ClinicalTrials.gov under the identifier: NCT04093180 on 17/09/2019.

## Introduction

Cerebral Palsy (CP) is a life-long group of disorders occurring in about 2 per 1000 live births [1]. CP is caused by non-progressive damage to the fetal or infant brain, which results in motor and postural disturbances, and leads to muscle spasticity, reduced functional abilities, and activity limitations [2].

Recent intensive rehabilitation methods combining upper and lower extremity training show promising results in children with CP [3]. Potential biomarkers of these positive findings are an increase in sensory and motor connectivity due to skilled, repeated movements during an intervention [4]. A crucial component of the studied intensive rehabilitation approaches is the application of goal-directed activities during the intervention, representing the principles of motor learning feedback and neuroplasticity [5-7]. Meanwhile, plastic changes in the developing brain tend to be stimulated by repetitive tasks with gradual complexity [8].

Constant practice of specific movements may enable automatization and shift motor control to memory-based processing by restructuring the cortical representations of sensorimotor features. This restructuring is believed to appear after intensive repetitive training in adult survivors with stroke and pediatric participants with CP [9]. Although changes in body structures or functions due to the neuroplastic modifications do not necessarily correlate with improvement in activity or participation, the last findings also highlighted that intensive motor training contributes to the child's well-being during their daily life [10]. Thus, many research groups focus on further development and understanding of the use of intensive repetitive motor training for children with CP [11].

Intensive Neurophysiological Rehabilitation System (INRS) includes intensive repeated procedures in which the difficulty of required movements gradually increases [12]. Interventions aim to improve different functions, influencing various pathogenic pathways and reaching a more considerable total effect by potentiating each other. Components consider the motivational aspect of rehabilitation and focus on the functional performance of daily life activities along with postural control and locomotion improvement. Unimanual and bimanual age-appropriate, goal-oriented activities for training fine and gross motor functions are delivered in a child-friendly way with elements of play. Treatment components of the INRS address different functional goals in the Body functions (joint mobility, muscle tone, voluntary movement, pain) and Activities and Participation (fine hand use, walking, moving around, interpersonal interactions, and family relationships) domains of the International classification of functioning, disability, and health (ICF) [13].

A retrospective analysis of treatment with INRS demonstrated a decrease in muscle tone, an increase in passive range of motion, and an enhancement of gross motor skills and fine motor function in most children with CP who received one course of INRS rehabilitation [14]. There were also several experimental studies of the INRS. A single-blind pre-post trial revealed an improvement in gross motor functions, an increase in passive range of motion (PROM) in the lower extremities, and a reduction of muscle spasticity after the two-week course with INRS [15]. According to recent research, enhancing lower extremity function is especially important because it correlates with the reduction in the severity of CP, resulting in a more significant number of children with the potential to walk [16]. There also was a before-after experimental study to assess the changes in hand function after the INRS course. The study indicated an increased dexterity of both hands and unimanual functions of the dominant hand in children with CP after the two-week treatment course with the INRS [17].

We formulated a hypothesis that rehabilitation according to INRS is superior to the standard home treatment for the functional abilities of children with CP. In this quazi-randomized controlled study, we aimed to study the effects of INRS treatment on upper- and lower-extremity functions, mobility, and self-care in the population of children with bilateral CP.

## Methods

### Study design

**Fig. 1:**
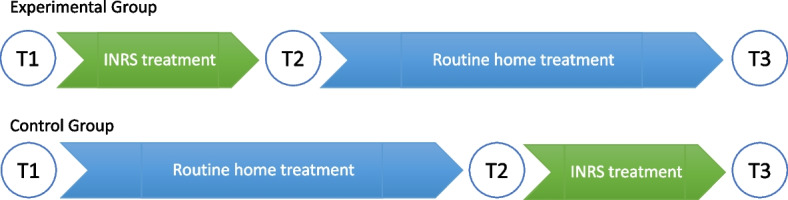
Timeline of the interventions and assessment procedures

A quasi-randomized, waitlist-controlled, assessor-blinded trial with two groups was conducted. Both groups received an INRS treatment course and four weeks of routine home treatment but in reverse order. The experimental group underwent INRS treatment and, after that, continued home treatment for four weeks. The control group stayed on the waiting list for four weeks receiving routine home treatment after the first evaluation and then came for INRS treatment (Fig. 1).

Patients who planned to receive treatment in the tertiary care facilities providing INRS treatment (International Clinic of Rehabilitation or Elita Rehabilitation Center) were considered potential participants. Candidates were selected according to defined inclusion criteria by reviewing their previously obtained medical documentation. The study coordinator remotely (via phone and e-mail) explained the study details to the family and child and re-checked the inclusion–exclusion criteria. If the child and parents were ready to participate and signed the informed consent form, they were randomly assigned to the experimental or control group by the study coordinator, that was not involved in the assessment or treatment processes.

Group allocation was performed using quasi-randomization whereby participants were assigned to the experimental or control group in the order in which they enrolled in the study in the step of 3 (the first 3 participants were assigned to the experimental group, then the next 3 to the control group, then next 3 to the experimental and so on).

All the participants were evaluated three times: at baseline -time T1, after the first period – time T2 (after INRS treatment for the experimental group and routine home treatment for the control group), and in the end – time T3 after groups switched treatments. Evaluations have been performed by certified and trained therapists blinded to group allocation. All data were forwarded to the supervisor on the same day. The study protocol was registered on ClinicalTrials.gov under the identifier: NCT04093180 on 17/09/2019.

### Participants

**Fig. 2:**
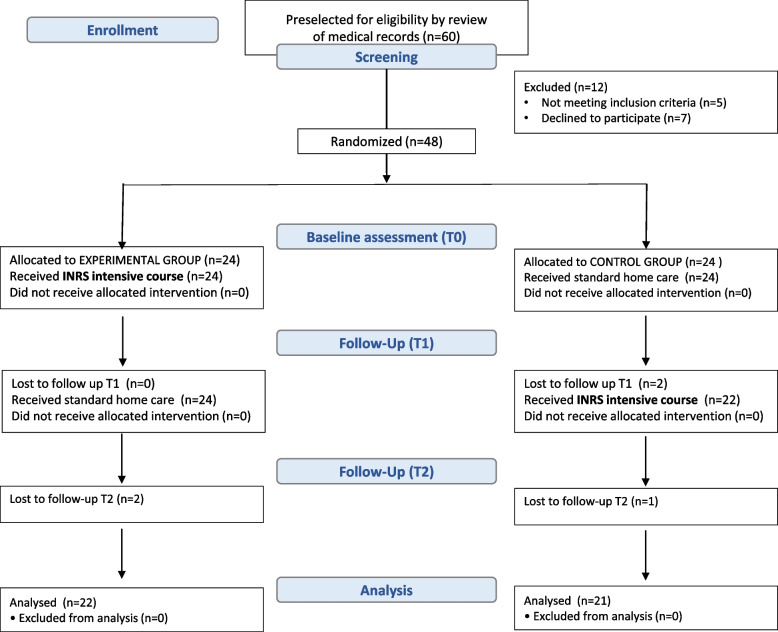
Patient flow

Participant flow is presented in the CONSORT flowchart (Fig. 2). Sixty children were preselected as possible participants in the study and underwent the primary screening. Further, 12 children have been excluded from the study due to non-compliance with the study requirements.

Forty-eight children were allocated to one of the groups using quasi-randomization with step 3 until both groups had 24 participants. Two patients were lost to follow-up during home treatment in the experimental group, and one child was excluded due to illness in the control group.

The demographic characteristics of the participants are presented in Table 1. The experimental group included 22 patients, and the control group – 21 children. There was no statistical difference between the groups.

**Table 1:** Demographic characteristics of the participants

Variable /characteristic	Experimental group	Control group
**Number of children**	22	21
**Age in years mean (SD)**	11.5 (3,1)	10.8 (3.3)
**Sex ratio (male/female)**	12/10	14/7
**Diagnosis**		
**CP: spastic quadriplegia**	18	17
**CP: spastic diplegia**	4	4
**GMFCS**		
**Level I**	8	7
**Level II**	11	10
**Level III**	3	4
**MACS**		
**Level I**	7	6
**Level II**	10	11
**Level III**	5	4

*SD* Standard deviation, *CP* Cerebral Palsy, *GMFCS* Gross Motor Functions Classification System, *MACS* Manual Abilities Classification System

 Inclusion criteria were: (i) CP, spastic bilateral forms (diagnosis based on the recommendations of the Surveillance of Cerebral Palsy in Europe [18]; (ii) 5 to 12 years of age; (iii) Gross Motor Function Classification System—Levels I-III; (iv) Manual Ability Classification System – Levels I-III. Exclusion criteria were: (i) uncontrolled seizures; (ii) severe intellectual disability; (iii) uncooperative behavior; (iv) surgery and Botox injections during the ongoing year.

### Intervention

We used the Template for Intervention Description and Replication TIDieR guide to describe Intensive Neurophysiological Rehabilitation System (INRS), also called Professor Kozyavkin Method [19].

The aim of the INRS is to improve the functioning and quality of life of children with CP by enhancing their mobility and self-care functions. Children received an intensive course of treatment, according to INRS, for two weeks. Details of the INRS program were individualized depending on each child's abilities, but, in general, the intervention was not modified during the study.

It included:

-30 min of physical therapy aimed at gross motor training is performed daily and includes task-related personalized gross motor exercises, including bimanual activities, postural skills practicing, endurance, mobility, and balance training,
-20 min of occupational therapy focused on developing skills necessary for the performance of everyday activities, including play and self-care imitated activities such as tying shoelaces, buttoning, threading beads, and games with the spiky ball,
-5 min of spinal manipulative therapy: a variation of the spinal manipulation carried out in lumbar, thoracic, and cervical regions using high-velocity, low-amplitude thrusts techniques,
-60 min of full body massage with reflexotherapy using different massage techniques together with isotonic and post-isometric relaxation techniques and applying low current electric stimulation of acupuncture points and myofascial trigger points, reinforced with wax and paraffin application
-15 min of joint mobilization when the physical therapist pulls the large and peripheral joint smoothly beyond the range of passive movements by applying pressure to the tissues surrounding the joint,
-20 min of computer game therapy when the child is training the movements needed in a functional context with special personalized games; the child can control the game character by moving their body and using special equipment, such as a motion-sensing input device for upper extremities training, dance mat for stepping games, and balance board for balance training,
-15 min of gait training on a treadmill with/without suspension,
-15 min of strength training (called "mechanotherapy") when muscle strength, endurance, flexibility, and posture are endorsed by applying special devices and technologies, such as suspension-based physical therapy, motorized movement therapy, and cycling,
-30 min of group rhythmic gymnastics: adapted active games and dancing movements that are performed in a group of peers and families.

More details about INRS are presented in the manual [12]. INRS treatment was provided by certified medical doctors, physical therapists, and medical nurses experienced in working with children with CP. It was provided individually, face to face, with one specialist per child. In the case of group rhythmic gymnastics, there are two staff members for the group. Rehabilitation was provided in the tertiary care facility for about four hours daily for ten days.

The medical staff supervisor has assessed the quality of every component delivery. No adverse events have been reported during the intervention.

### Outcome measures

Certified therapists blinded to group allocation measured all outcomes three times. The assessments covered the three domains of ICF.

The primary outcome measure was Gross Motor Function Measure 66 Item Set (GMFM), which has good reliability and responsiveness in children with CP [20]. It uses four targeted item sets to evaluate overall gross motor ability in children with CP under five dimensions. The administration of the GMFM starts with a predefined decision item. The child's score on each item leads therapists to the item set most suitable for that child.

The secondary outcome measures included the Jebsen-Taylor Hand Function Test, Box and Blocks Test, ABILHAND-Kids Questionnaire, Self-care and Mobility domain of the Pediatric Evaluation of Disability Inventory, and the ankle dorsiflexion passive range of motion.

The Jebsen-Taylor Hand Function Test assesses hand function activities during the performance of activities of daily living [21]. The test quantifies the time it takes for the subject to do the following standardized functional tasks with one hand: turning over cards, picking up small items, simulating feeding, stacking checkers, picking up light cans, and picking up heavy cans. Guidelines specify that testing begins with the non-dominant hand. The writing task was excluded from the assessment due to the age of some participants. Each item is scored according to the time to complete the task. The scores for all items are then summed for a total score for each hand.

Box and Blocks Test is a valid and reliable diagnostic tool that evaluates the level of manual dexterity [22]. The score is the number of blocks the dominant and non-dominant hands carry from one compartment to another in one minute.

ABILHAND-Kids measures the manual ability of children with upper limb impairment [23]. The scale measures a person's ability to manage daily activities that require manual ability. The parent is asked to fill in the Ukrainian version of the questionnaire by estimating their child's performance in 21 manual activities on a 3-level scale (impossible, difficult, easy) [24]. A total score is calculated and presented in the logits (the linear measure that expresses the odds of success of the patient in performing tasks).

The Pediatric Evaluation of Disability Inventory (PEDI) is an instrument that measures independence in daily living. It covers daily activities in self-care, mobility, and social functioning among children with CP [25]. For our study, we used self-care and mobility domains. The scaled score was determined using the raw score.

The passive range of ankle dorsiflexion (PROM) was measured with a hand-held goniometer. For each child, we measured both the left and right sides with the knees flexed. A standardized assessment protocol was followed for positioning the patient and the examiner's hand [26].

### Sample size

The sample size was calculated based on our previous study of changes in motor functions in children with CP after the course of Intensive Neurophysiological Rehabilitation [16]. A mean improvement of the GMFM score from 58.8 to 60.2 with a mean difference (MD) of 1.4 ± 2.9 points was reported. With a 5% of probability of a type I error (α = 0.05) and 80% power to detect a possible difference (1-β = 0.08), a minimum of 20 participants per group was required. Considering drop-ups during the study, 24 children were allocated to each group.

### Statistics

Statistical analysis was performed using SPSS ver. 23 [27]. A null hypothesis rejection was set at *p* < 0.05 for all measurements. Appropriate statistical assumptions of normality and variance homogeneity for each general linear model were tested using the Shapiro–Wilk test before hypothesis testing.

To evaluate changes over time for each variable, the repeated measures analysis of variance tests (ANOVA) with Bonferroni posthoc was performed separately for each group. The effect size was estimated using the value Partial Eta Squared (η2). Values of η2 = 0.01 indicate a small effect; η2 = 0.06 indicates a medium effect; η2 = 0.14 indicates a large effect.

In case of statistically significant change during the treatment in the experimental group, these changes have been compared with the same period (between time T1 and time T2) in the control group using the Analysis of Covariance (ANCOVA) test with time T1 values as a covariate. The effect size was also calculated using η2.

## Results

**Fig. 3:**
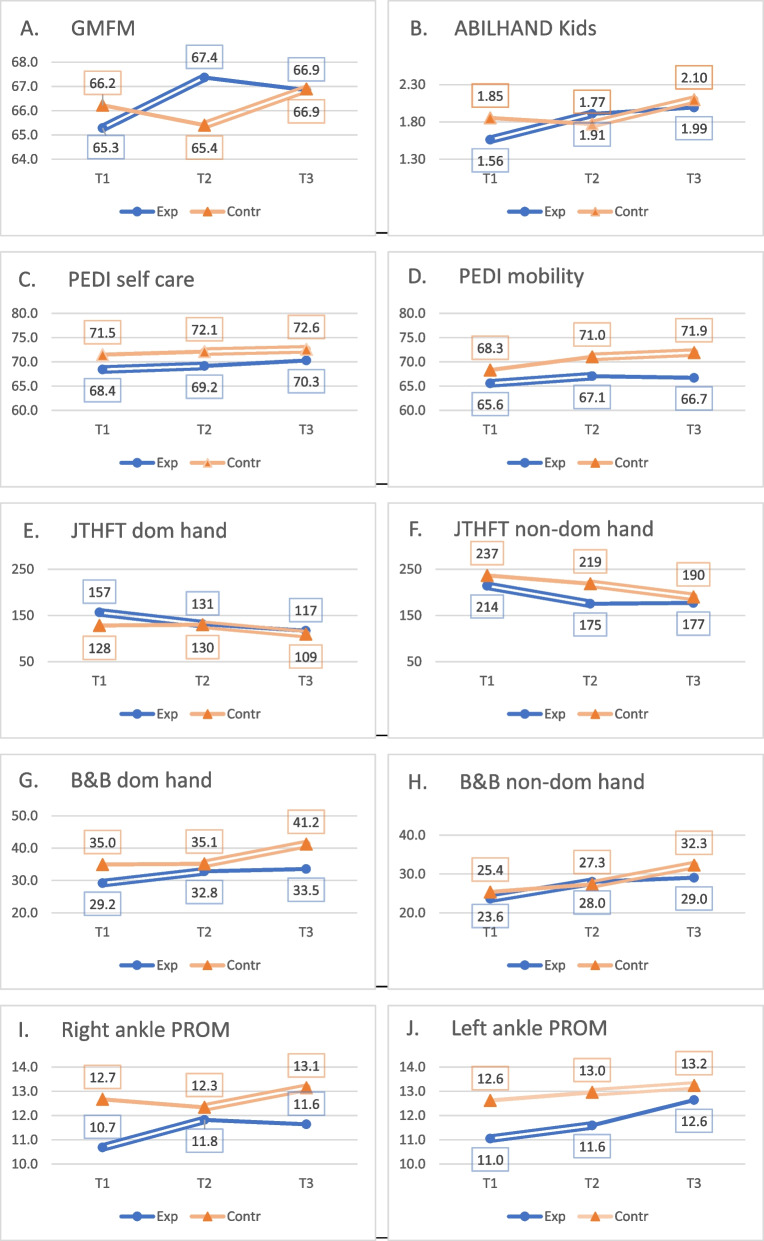
Chart of study results

All the study results are presented in Table 2 and Fig. 3. Repeated measures ANOVA was used to verify whether there was a statistically significant difference between the means of three measurements (Time T1, Time T2, and Time T3).

**Table 2:** Comparison of outcome measures for the experimental and control group

				*Within-group changes repeated measures ANOVA*		*Between-group difference ANCOVA*	
	*Time T1 mean (SD)*	*Time T2 mean (SD)*	*Time T3 mean (SD)*	*df; F sign difference*	*significance (p); effect size (η2)*	*Mean difference df; F*	*significance (p); effect size (η2)*
**GMFM**							
Exp. group	65.3 (15.4)	67.3 (15.6)	66.8 (15.2)	df = 2,42; F = 5.85 T1 ≠ T2, T1 ≠ T3	***P* < 0.01** η2 = 0.21	**2.89** df = 1,40 F = 6.03	***P* < 0.01** η2 = 0.12
Contr. group	66.2 (13.1)	65.4 (12.8)	66.9 (14.4)	df = 2.40; F = 3.25 T2 ≠ T3	***P* < 0.05** η2 = 0.14		
**ABILHANDS Kids**							
Exp. group	1.56 (2.1)	1.91 (2.0)	1.99 (2.2)	df = 2,42; F = 2.50	*P* = 0.07		
Contr. group	1.85 (1.8)	1.77 (1.2)	2.10 (0.9)	df = 2,40; F = 2.36	*P* = 0.09		
**PEDI self-care scale**							
Exp. Group	68.4 (12.2)	69.2 (11.2)	70.3 (12.3)	df = 2,42; F = 2.21	*P* = 0.1		
Contr. Group	71.5 (9.8)	72.1 (8.9)	72.6 (8.2)	df = 2,40; F = 0.83	*P* = 0.4		
**PEDI mobility scale**							
Exp. Group	65.6 (16.8)	67.1 (14.8)	66.7 (15.7)	df = 2,42; F = 0.50	*P* = 0.631		
Contr. Group	68.3 (11.8)	71.0 (11.9)	71.9 (22.1)	df = 2,40; F = 2.2	*P* = 0.12		
**JTHFT DH**							
Exp. Group	156.6 (88.8)	131.1 (92.7)	116.8 (89.7)	df = 2,42; F = 11.1 T1 ≠ T2, T1 ≠ T3	***P* < 0.01** η2 = 0.345	**25.1** df = 1.40 F = 6.22	***P* < 0.01** η2 = 0.18
Contr. Group	128.3 (77.4)	129.9 (87.7)	109.5 (71.1)	df = 2,40, F = 3.14 T2 ≠ T3, T1 ≠ T3	***P* < 0.05** η2 = 0.14		
**JTHFT NDH**							
Exp. Group	214.2 (147)	175.2 (141)	177.0 (142)	df = 2,42; F = 6.30 T1 ≠ T2, T1 ≠ T3	***P* < 0.01** η2 = 0.24	**23.8** df = 1.40 F = 4.32	***P* < 0.05** η2 = 0.11
Contr. Group	236.6 (105)	218.5 (108)	190.3 (116)	df = 2,40, F = 8.03 T2 ≠ T3, T1 ≠ T3	***P* < 0.01** η2 = 0.27		
**B&B DH**							
Exp. Group	29.2 (12.3)	32.8 (14.0)	33.5 (16.1)	df = 2,42; F = 7.45 T1 ≠ T2, T1 ≠ T3	***P* < 0.01** η2 = 0.24	**3.17** df = 1.40 F = 4.08	***P* < 0.05** η2 = 0.09
Contr. Group	35.0 (9.7)	35.1 (8.6)	41.2 (12.0)	df = 2,40, F = 7.33 T2 ≠ T3, T1 ≠ T3	***P* < 0.01** η2 = 0.21		
**B&B NDH**							
Exp. Group	23,6 (11,2)	28,0 (15,0)	29,0 (16,8)	df = 2,42; F = 8.3 T1 ≠ T2, T1 ≠ T3	***P* < 0.01** η2 = 0.26	**2.44** df = 1.40 F = 3.77	***P* < 0.05** η2 = 0.07
Contr. Group	25,4 (10,9)	27,3 (9,6)	32,3 (10,6)	df = 2,40, F = 7.38 T2 ≠ T3, T1 ≠ T3	***P* < 0.01** η2 = 0.22		
**Right Ankle PROM**							
Exp. Group	10.7 (4.8)	11.8 (4.8)	11.6 (3.5)	df = 2,42; F = 1.48	*P* = 0.24		
Contr. Group	12.7 (4.0)	12.3 (2.5)	13.1 (2.3)	df = 2,40, F = 0.49	*P* = 0.61		
**Left Ankle PROM**							
Exp. Group	11.0 (4.8)	11.6 (4.5)	12.6 (3.7)	df = 2,42; F = 2.04	*P* = 0.14		
Contr. Group	12.6 (2.7)	13.0 (2.3)	13.2 (2.6)	df = 2,40, F = 0.29	*P* = 0.74		

*SD* Standard deviation, *df* degrees of freedom, *DH* dominant hand, *F* F ratio (residual variance), *η2* partial eta-squared (measures effect size), *NDH* non-dominant hand

GMFM score was the primary outcome measure (Fig. 3A). In the experimental group, the baseline GMFM value (T1) was significantly different from the post-INRS treatment score (T2); it increased by 2.0 points (67.3–65.3 = 2.0, *P* < 0.01). To measure the effect size in our ANOVA model, we used η2. Obtained values of η2 = 0.21 indicated a large effect size. There was no significant difference after the home treatment (T2-T3). There were no significant changes for the control group during routine home treatment (period T1-T2), but after INRS treatment, the GMFM score increased by 1.5 points (66.9–65.4 = 1.5). This change was statistically significant (*p* < 0.05) with a large size effect (η2 = 0.14). The difference between groups during the first study period (INRS treatment for the experimental group and routine home treatment for the control group) was assessed using the ANCOVA statistics with baseline values (time T1) as a covariate. ANCOVA statistics were calculated only if a statistically significant difference was observed in at least one of the groups. Data indicated the superiority of the INRS treatment compared with routine home treatment in GMFM score change. MD of 2.89 points was statistically significant (*p* < 0.01) with a medium effect size (η2 = 0.12).

Changes in the ABILHANDS-Kids score were not statistically significant in both groups but were close to the significance threshold (Fig. 3B). After the INRS treatment in the experimental group, the ABILHANDS-Kids score improved by 0.35 logits with *p*-values equal to 0.07.

PEDI self-care scale scores revealed no statistically significant change in both groups (Fig. 3C). The changes in the PEDI mobility scale were also not significant in both groups and after both treatment periods (Fig. 3D).

The Jebsen-Taylor Hand Function Test (JTHFT) and Box and Blocks Test (BBT) were secondary outcome measures aimed at hand function assessment, performed separately for the dominant and non-dominant hand. Results of the JTHFT of the dominant hand are presented in Table 2 and Fig. 3E. In the experimental group, after the INRS treatment, the JTHFT time needed to perform all the tasks decreased by 24 s (156 -131 = 24). This change was statistically significant (*P* < 0.01) with a large size effect (η2 = 0.34). Changes during routine home treatment were insignificant. The control group had no statistically significant changes after INRS and during home treatment. The difference between groups during the first study period (INRS treatment for the experimental group and routine home treatment for the control group) was statistically significant (the difference of the means was 25.1 points) with a large effect.

Similar results were obtained during the assessment of the non-dominant hand (Fig. 3F). In both groups, we observed a statistically significant decrease in JTHFT score after the INRS treatment and non-significant changes during routine home therapy. Also, there was a statistically significant difference (*p* < 0.05) between groups, with MD of 23.8 points and a medium-size effect (η2 = 0.11).

Results of the Box and Blocks (B&B) Test indicated statistically significant improvement after INRS treatment for both dominant and non-dominant hands (Table 2, Fig. 3G, H). During home treatment, changes were not significant. The between-group difference for the dominant hand was 3.17 points (*p* < 0.05) with medium size effect (η2 = 0.09). For the non-dominant hand, the between-group difference was 2.44 (*p* < 0.05) with a medium-size effect (η2 = 0.07).

The passive range of ankle dorsiflexion (PROM) of both legs was measured three times in both groups (Table 2, Fig. 3I, J). There was no statistically significant difference between measures in both groups.

## Discussion

The aim of this quasi-randomized controlled study was to assess the effects of the Intensive Neurophysiological Rehabilitation System on the population of children with bilateral CP.

We found an increase in gross motor function (GMFM), manual dexterity (BBT), and hand function (JTHFT) after the course of INRS in children with bilateral CP. At the same time, we did not find any significant changes in the scores of the PEDI self-care and mobility domains and the ABILHAND-Kids questionnaire.

The obtained results are consistent with previous experimental research on the effects of INRS on the gross motor function and hand function of children with CP. In the single-blind study of INRS, an increase in the GMFM-66 score was detected after one rehabilitation course [16]. Hand function, particularly dexterity of both hands and unimanual functions of the dominant hand, had improved in another pre-post study of INRS [17].

We suggest that our findings in motor function may appear due to the intensity, repetition, and functional components of the INRS intervention. Participants received intensive treatment for up to 4 h, five days a week, over a period of 2 weeks. And the current literature has reported positive mobility outcomes with treatment dosages ranging from 2 to 5 days a week for two weeks and more [28].

Other authors achieved similar results. An experimental study of intensive rehabilitation described concomitant changes in both upper and lower extremities in the population of children with bilateral CP [3]. Another prospective clinical study of the effects of intensive functional therapy revealed significant post-intervention improvement in hand function, mobility, and daily function of adolescents with CP [29]. Possible mechanisms of improvement in motor function are changes in motor and sensory connectivity and increased strength induced by motor learning during an intensive repetitive motor intervention [4, 30].

We observed positive but not statistically significant improvements in the outcome measures aimed at evaluating the changes in activities of daily living, such as the score of ABILHAND-Kids and PEDI assessment. The possible reason for this finding is that participants stayed in the rehabilitation center during their experimental treatment, and children did not have the pre-conditions to undertake their typical activities. Another rationale for no detectable changes in activities of daily living is the short follow-up; the average duration of follow-up in other studies was at least three months [3, 29], and this time seems more appropriate to detect changes in daily life by caregivers.

This study has several limitations. The main limitation is the absence of classic randomization because we could not implement it methodologically. Instead, we applied quazi-randomization. Another limitation is the follow-up at one month. In other studies, excellent progression appeared at the follow-up at three months [3]; but our patients come for intensive rehabilitation from the whole country, and the longer follow-up would lead to a higher drop-out rate; therefore, we decided to establish one month as a follow-up. Another peculiarity of our study is a potential therapist effect that can appear because many different health professionals were involved in the intervention. The same professionals provided the treatment without substitution for every participant to eliminate this limitation.

This quazi-randomized controlled trial also has noticeable strengths. This study is the first assessor-blinded trial with two groups that evaluates the effects of INRS on different functions in children with CP. The findings of this research allow for planning further studies with a longer follow-up that potentially may facilitate exploring changes in the Activities and Participation domains of ICF. Moreover, it added evidence to understanding how gross and fine motor skills improve after short-term intensive rehabilitation in children with bilateral CP in their middle childhood.

## Conclusions

Study indicates that intensive rehabilitation with INRS improves gross motor and hand function in children with bilateral CP. In further studies, we plan to focus on the longer follow-up to assess the changes in the activities of daily life and participation level.

## Data Availability

The primary data generated or analyzed during this study are included in this published article. More details regarding data used and analysed during the current study are available from the corresponding author on reasonable request.

## Abbreviations

η2: Partial eta squared
ANCOVA: Analysis of covariance
ANOVA: Analysis of variance
BBT: Box & Blocks Test
CP: Cerebral palsy
GMFCS: Gross Motor Function Classification System
GMFM: Gross Motor Function Measure 66 Item Set
ICF: International Classification of Functioning, Disability, and Health
INRS: Intensive Neurophysiological Rehabilitation System
JTHFT: Jebsen-Taylor Hand Function Test
MACS: Manual Ability Classification System
MD: Mean difference
PEDI: Pediatric Evaluation of Disability Inventory
PROM: Passive range of motion
SD: Standard deviation
